# Cutaneous effects of ovatoxin-a: an in vitro study on human skin keratinocytes

**DOI:** 10.1007/s00204-025-04229-3

**Published:** 2025-10-31

**Authors:** Alessandra D’Arelli, Michela Carlin, Silvio Sosa, Chiara Melchiorre, Fabio Varriale, Luciana Tartaglione, Michela Varra, David Kulis, Donald M. Anderson, Mark Poli, Aurelia Tubaro, Carmela Dell’Aversano, Marco Pelin

**Affiliations:** 1 Department of Life Sciences, University of Trieste, Trieste, Italy; 2 School of Medicine and Surgery, Department of Pharmacy, University of Naples Federico II, Naples, Italy; 3 NBFC, National Biodiversity Future Center, Palermo, Italy; 4 WHOI, Woods Hole Oceanographic Institution, Woods Hole, MA 02543 USA; 5 Diagnostic Systems Division, USAMRIID, U.S. Army Medical Research Institute of Infectious Diseases, Ft Detrick, MD USA

**Keywords:** Ovatoxin-a, *Ostreopsis* cf. *ovata*, Palytoxin, Cutaneous toxicity, Mechanism of action

## Abstract

Ovatoxin-a (OVTX-a) is the main palytoxin (PLTX) analogue produced by the marine benthic dinoflagellate *Ostreopsis* cf. *ovata* from the Mediterranean area, where it poses risks for human health mainly through inhalation of marine aerosols and/or skin contact with contaminated seawater. Herein, we highlight the effects of OVTX-a, in comparison to PLTX, at the skin level using human HaCaT keratinocytes, employing a sample containing OVTX-a at 86% purity. After 4 h exposure, OVTX-a reduced cell viability (EC_50_ = 8.3 × 10^−9^ M) with one order of magnitude lower potency than PLTX (EC_50_ = 3.7 × 10^−10^ M). Accordingly, OVTX-a induced lower levels of cell necrosis and mitochondrial depolarization than PLTX, but a 1-h-exposure to each toxin provoked similar increases in ROS production. Moreover, cells co-exposure to ouabain, an inhibitor of Na^+^/K^+^ ATPase, the molecular target of PLTX, or to diphenyliodonium chloride, a non-specific inhibitor of flavoprotein-based enzymes involved in PLTX-induced oxidative stress, suggested that OVTX-a and PLTX share not only the same molecular target, but also the same mechanism of ROS-dependent cytotoxicity. Overall, these results provide new evidence on OVTX-a hazard characterization at the skin level, clarifying its mechanism of action at the basis of cutaneous adverse effects experienced by bathers during *O.* cf. *ovata* blooms.

**Supplementary Information:**

The online version contains supplementary material available at 10.1007/s00204-025-04229-3.

## Introduction

Ovatoxin-a (OVTX-a) is a structural analogue of palytoxin (PLTX), a well-known marine non-proteinaceous toxin, initially isolated from zoanthids belonging to the genus *Palythoa* (Moore and Scheuer 1971). Subsequently, PLTX and its congeners have also been identified in other marine organisms, including benthic dinoflagellates of the genus *Ostreopsis*. Ovatoxins (OVTXs) are the main PLTX analogues found in *Ostreopsis* cf. *ovata* and *O. fattorussoi* from the Mediterranean Sea (Ciminiello et al. 2010, 2012; Accoroni et al. 2016; Açaf et al. 2020; Chomérat et al. 2022). Since their discovery in the Gulf of Thailand, these unicellular microalgae are typically distributed in tropical and sub-tropical areas, especially in the Pacific, Atlantic and Indian Ocean (Faust et al. 1996; Rhodes 2011; Gu et al. 2022). However, probably as a result of global climate change and water warming, their presence has been reported also in temperate areas, such as the Mediterranean Sea (Pavaux et al. 2020; Tester et al. 2020), where their occurrence has been documented. Among them, *O*. cf. *ovata* seems to be the predominant *Ostreopsis* species in the Mediterranean Sea, where it appears to produce mainly OVTX-a, as the principal component of the toxin profile, together with other structural analogues (OVTX-b to -h) and traces of a structural isomer of PLTX, named isobaric PLTX (Tartaglione et al. 2017). OVTX-a has been detected in different components of the marine food web, including mussels, sea urchins and fish from several Mediterranean coastal sites, even if no human poisonings related to consumption of OVTX-contaminated seafood have been reported so far (Aligizaki et al. 2008; Amzil et al. 2012; Biré et al. 2015; Carella et al. 2015; Neves et al. 2018; Sardo et al. 2021; Accoroni et al. 2022). Several cases of adverse effects in humans associated with cutaneous and/or inhalation exposure to seawater or marine aerosols have been recorded concomitantly with *O.* cf*. ovata* blooms since their first appearance along the French coasts of Villefranche-sur-Mer in the early 1970s (Ciminiello et al. 2014). In particular, *O.* cf. *ovata* has been blooming repeatedly and massively along the coasts of Italy, Spain, France and Monaco, causing serious human suffering (Zingone et al. 2006; Barroso García et al. 2008; Kermarec et al. 2008; Tichadou et al. 2010). The most reported clinical picture in poisoned patients included respiratory symptoms such as rhinorrhea, cough, dyspnea and/or bronchoconstriction, conjunctivitis, and signs of skin inflammation (Durando et al. 2007; Barroso García et al. 2008; Tichadou et al. 2010; Tubaro et al. 2011). In concomitance with massive toxic outbreaks occurred along the Ligurian coasts (North-Western Italy) in 2005 and 2006, *O.* cf. *ovata* was identified as a producer of PLTX congeners (Ciminiello et al. 2006) and OVTX-a was identified for the first time as the likely causative agent of the clinical symptoms (Gallitelli et al. 2005; Brescianini et al. 2006; Ciminiello et al. 2006; Durando et al. 2007). Despite the impact of OVTX-a on human health, studies characterizing the hazard posed by this toxin are very limited due to the challenges in obtaining well-characterized and accurately-quantified OVTX-a from *O.* cf. *ovata* cultures at a sufficient purity to carry out toxicological evaluations ^29^.

In a previous study on HaCaT keratinocytes, we highlighted a difference between the cytotoxic potency of OVTX-a and PLTX, showing that OVTX-a was around 100-fold less cytotoxic than PLTX. These results were obtained using the commercially available non-certified standard of PLTX from *P. tuberculosa* (lot number WKL7151, purity > 90%) and a sample of OVTX-a isolated from the Adriatic *O.* cf. *ovata* strain OOAN0816 (Ciminiello et al. 2012) containing 51% OVTX-a, 12% OVTX-d, 25% OVTX-e, as well as assorted isobaric PLTX and ovatoxins, accounting each for 0.4 to 6.3% of the total sample content (Pelin et al. 2016b). Cytotoxicity results obtained on this sample (Pelin et al. 2016b) have not been fully confirmed by those subsequently obtained by other authors testing OVTX-a on a panel of cell lines, including HaCaT cells, where OVTX-a showed a slightly lower, still similar, cytotoxicity than PLTX (Gémin et al. 2022; Lanceleur et al. 2024). In addition, a previous in vivo toxicity study in rats showed slight, but not significant, differences in the lethal potency of OVTX-a and PLTX following inhalational exposure (median lethal dose, LD_50_ = 0.031 and 0.041 μg/kg, respectively) or intraperitoneal injection (LD_50_ = 3.26 and 1.81 μg/kg, respectively) (Poli et al. 2018). Thus, there is an urgent need to carefully characterize the hazard posed by OVTX-a using a high purity grade toxin.

Hence, the in vitro study reported herein aims to investigate cutaneous effects induced by a recently isolated sample of OVTX-a (purity 86%) on human skin HaCaT keratinocytes, in comparison to those of PLTX standard, used as reference compound, also exploring whether the two toxins share the same mechanism of action. 

## Materials and methods

### Chemicals

PLTX from *Palythoa tuberculosa* was purchased from Wako Pure Chemicals Industries Ltd. (Osaka, Japan; Lot n° CDQ7083, purity: > 90%). OVTX-a was isolated from *Ostreopsis* cf. *ovata* cultures as described below. All the other reagents of analytical grade were purchased from Sigma-Aldrich (Milan, Italy), if not differently specified. Methanol (MeOH) HPLC grade ≥ 99.9%, ethanol (EtOH) HPLC grade > 99.7%, acetonitrile (ACN) HPLC grade ≥ 99.9%, water (W) HPLC grade, and glacial acetic acid (AcOH) for HPLC were all from VWR International Srl (Milan, Italy). 2-propanol (2-PrOH) HPLC grade ≥ 99.9% was from Merck KGaA (Darmstadt, Germany).

### *Ostreopsis* cf. *ovata* culture as starting material

*Ostreopsis* cf. *ovata,* isolate NIES3351, was established from a water sample collected at Issai Otsuki, Kochi Prefecture, Japan on August 8th, 2009. This OVTX-producing culture was purchased from the National Institute of Environmental Studies (NIES) Culture Collection (Tsukuba, Japan). Cultures were grown in f/6-Si (without silicate) media. ^34^ Here, all the nutrient additions were reduced by a factor of 3 compared to f/2, and the trace metals were further modified as described in Anderson et al. (Anderson et al. 1994) through the addition of Na_2_SeO_3_ and the reduction of the concentration of CuSO_4_·5H_2_O, each to final concentrations of 10^–8^ M. The medium was prepared using sterile 0.2 µm filtered, UV-irradiated, Vineyard Sound (Cape Cod, MA) seawater (salinity ~ 32). *Ostreopsis* stock, seed and production cultures were maintained at 23 °C on a 14:10 h light:dark cycle (ca. 250 µmol photons⋅m^2^/sec irradiance provided by cool white fluorescent bulbs). Further details are reported in Supplementary materials (Method S1).

### OVTX-a extraction, purification and quantitation

Isolation of OVTX-a was carried out on the algal pellet obtained from 51 L of *O.* cf *ovata* NIES3351 culture containing approximately 8 × 10⁶ cells/L by a 4-step procedure, including Extraction, Medium Pressure Liquid Chromatography (MPLC), and two preparative High Performance Liquid Chromatography (HPLC) purification steps, recently further optimized by Miele et al. (2024).

The MPLC fractions richest in OVTXs were pooled (2.6 L), concentrated under N_2_ stream at room temperature with ACN added during the concentration process up to a final volume of 2 mL ACN:W 2:8 (v/v) and underwent HPLC-step 1 under the experimental previously reported (Miele et al. 2024). The semi-purified fraction (300 mL), containing all the OVTXs was concentrated at room temperature under N_2_ stream to 2.0 mL and then subjected to HPLC-step 2, to separate OVTX-a from the other congeners, under the experimental conditions previously described(Miele et al. 2024). Further details are reported in Supplementary materials (Method S2).

#### LC-HRMS analyses

LC–HRMS analyses were conducted by using a hybrid linear ion trap LTQ Orbitrap XL™ Fourier Transform Mass Spectrometer (FTMS), equipped with an ESI ION MAX™ (SN 01719B) combined with a Dionex Ultimate 3000 HPLC system (Thermo-Fisher, San Jose, CA, USA), equipped with a quaternary pump (SN 8,099,775), thermostatic autosampler (SN 8,100,164) with a 100 μL sample loop, and a column oven (SN 6,004,692). Full scan HRMS spectra were acquired in positive ion mode within the mass range *m/z* 800–1400 at a resolving power (RP) 60,000 (FWHM at *m/z* 400). ESI sources parameters were: spray voltage 4.8 kV, capillary temperature 360 °C, capillary voltage 36 V, sheath gas 60 and auxiliary gas 21 (arbitrary units), and tube lens voltage 100 V. Extracted Ion Chromatograms (XICs) for each OVTX were obtained by selecting the exact mass of the mono-isotopic ion and the most intense ion peaks of the [M + H + Ca]^3+^ ion cluster of each congener from total ion current of the Full-scan HRMS experiment (Supplementary Table S1). Elemental formulae were assigned by Thermo Xcalibur software v2.2 SP1.48 (Thermo Fisher, San Josè, CA, USA) within a mass tolerance of 5 ppm. Quantitation was performed using a single and multiple level external calibration approach. In the absence of OVTX-a certified reference material, a five-point calibration curve of PLTX from Wako Chemical diluted in EtOH:W (1:1, v/v) at 1000, 500, 250, 125, and 62.5 ng/mL (y = 5 × 10⁶x + 103,942; R^2^ = 0.9982) was employed, assuming a comparable molar response between PLTX and OVTX-a based on their structural similarity. All quantitative analyses throughout the purification procedure were performed using the LC fast gradient while the purity of the final product was evaluated using the slow gradient #2 as previously described (Miele et al. 2024). Purity was assessed by LC-HRMS only, by limiting the observation to the PLTX-like compounds known to date in the mass range *m/z* 800–1400 (positive ions).

### Cell culture

HaCaT cell line was purchased from Cell Line Service (DKFZ; Eppelheim, Germany) and was cultured in Dulbecco’s Modified Eagle Medium supplemented with 10% fetal bovine serum, 1.0 × 10^−2^ M L-glutamine, 1.0 × 10^−4^ g/mL penicillin and 1.0 × 10^−4^ g/mL streptomycin at 37 °C under a humidified 95% air/5% CO_2_ atmosphere. Cell passage was performed 2 days post-confluence, once a week.

### Cell treatment

According to previous studies (Pelin et al. 2011, 2016a) the concentrations range of OVTX-a and PLTX selected for cells treatment was 1.0 × 10^−16^–1.0 × 10^−7^ M. Freshly-prepared working solutions were made in cell culture medium for each single experiment, starting from stock solutions (1.0 × 10^–6^ M in 50% EtOH).

For co-association experiments with ouabain (OUA) or diphenyleneiodonium chloride (DPI), concentrations of each inhibitor (1.0 × 10^−5^ M and 5.0 × 10^−6^ M, respectively) were selected based on previous studies on PLTX (Pelin et al. 2013b, a). After pre-exposure with each inhibitor for 1 h, cells were co-exposed with the same inhibitor and OVTX-a or PLTX for either 1 or 4 h.

### MTT assay

The effect of each toxin on HaCaT cell viability was assessed by the 3-(4,5-Dimethylthiazol-2-yl)-2,5-diphenyltetrazolium bromide (MTT) assay. Cells (5 × 10^3^ cells/well) were plated in 96-well plates for 72 h. After cell exposure to each toxin, medium was removed and replaced with fresh culture medium containing 0.5 mg/mL MTT. After 4 h, insoluble formazan crystals were solubilized with dimethylsulfoxide (DMSO; 200 μL/well) and the absorbance was measured at 540/630 nm, using the automated Microplate Reader FLUOstar® Omega (BMG LABTECH; Ortenberg, Germany). Data are reported as % of cell viability with respect to negative controls.

### LDH release assay

The toxins effect on cells membrane integrity and the resulting necrotic-like cell death was evaluated by the CytoTox 96® Non-Radioactive Cytotoxicity Assay (Promega; Madison, WI, USA) following manufacturer’s indications on HaCaT cells (5 × 10^3^ cells/well) cultured in 96-well plates for 72 h and then exposed to each toxin for 4 h. As positive control, cells were treated with Lysis 10X Solution [9% (v/v) Triton® X-100 in water], provided by the kit. The absorbance was measured at 490 nm by the automated Microplate Reader FLUOstar® Omega (BMG LABTECH; Ortenberg, Germany). Data were reported as % of cell death with respect to positive controls.

### Transmission electron microscopy (TEM) analysis

Cell were seeded in T25 flask at a density of 1 × 10^6^ cells/flask. After two days, cells were exposed to PLTX or OVTX-a (1.0 × 10^−9^ M) for 4 h. Subsequently, cells were detached by trypsinization, centrifuged, and resuspended in 2.5% glutaraldehyde (Electron Microscopy Sciences; Hatfield, PA, USA) in 0.1 M phosphate buffer. After 1 h, each sample was immersed in 0.1 M sodium phosphate buffer containing 1% osmium tetroxide for 1 h, at 4 °C. Afterwards, cells were dehydrated by a gradient of ethanol, followed by propylene oxide, and embedded in epoxy resin (Durcupan™ ACM). Ultrathin Sects. (70–90 nm), obtained by a UltraCut Microtome (Reichert-Jung; Depew, NY, USA), were contrasted with UranyLess (Electron Microscopy Sciences; Hatfield, PA, USA) and lead citrate (Electron Microscopy Sciences; Hatfield, PA, USA). Sections were analyzed using a Philips FEI EM208 transmission electron microscope and images were acquired using the digital camera QUEMESA and the RADIUS 2.0 EMSIS software.

### JC-1 assay

Mitochondrial depolarization was measured using the JC-1 mitochondrial staining kit following manufacturer’s instruction, after cells culturing (5 × 10^3^ cells/well) in 96-well plates for 4 days as previously reported(Pelin et al. 2014). As positive control cells were treated with 0.1 µg/mL valinomycin.

Fluorescence was detected by the automated Microplate Reader FLUOstar® Omega (BMG LABTECH; Ortenberg, Germany). Red fluorescence given by JC-1 aggregates, representing intact mitochondria, was measured at a 490/530 nm wavelength combination, while green fluorescence given by monomeric JC-1, representing disrupted mitochondria, at a 525/590 nm wavelength combination. Data were reported as % of mitochondrial depolarization with respect to negative controls.

### NBT reduction assay

The nitro blue tetrazolium chloride (NBT) reduction assay was used to assess ROS production on cells (15 × 10^3^ cells/well) cultured in 96-well plates for 4 days. Cells were then exposed to each toxin for 1 h in 0.5 mg/mL NBT in PBS containing 25 mM Hepes (200 µL/well) and the formed diformazan salts were solubilized with 140 µL DMSO and 120 µL 2 M KOH. As positive controls, cells were exposed to 1.0 × 10^−2^ M 2,2′-azobis(2-amidinopropane)-dihydrochloride (AAPH). The absorbance was measured at 630 nm using an automated Microplate Reader FLUOstar® Omega (BMG LABTECH; Ortenberg, Germany). Data are reported as % of ROS production with respect to negative controls.

### Statistical analysis

Results are presented as mean ± standard error of the mean (SEM) from at least three independent experiments performed in triplicate. Data was analyzed by a two-way analysis of variance (ANOVA), followed by Bonferroni’s test, or by Student’s *t*-test, using the GraphPad Prism software (version 10.3.1). Statistical differences were considered significative for *p* < 0.05.

## Results

### OVTX-a purification and characterization

Ovatoxin-a was isolated from an *O.* cf. *ovata* (NIES3351) culture using LC-HRMS to detect and quantify the toxin throughout the purification process. LC-HRMS analysis of the crude extract showed the presence of OVTX-a (73%), -d/-e (17%) and -isobaric PLTX (1%) as well as that of an unknown OVTX-congener (9%). Full scan HRMS spectrum of the crude extract and XICs of individual OVTXs are reported in Supplementary Information (Fig. S1, S2, respectively).

**Fig. 1:**
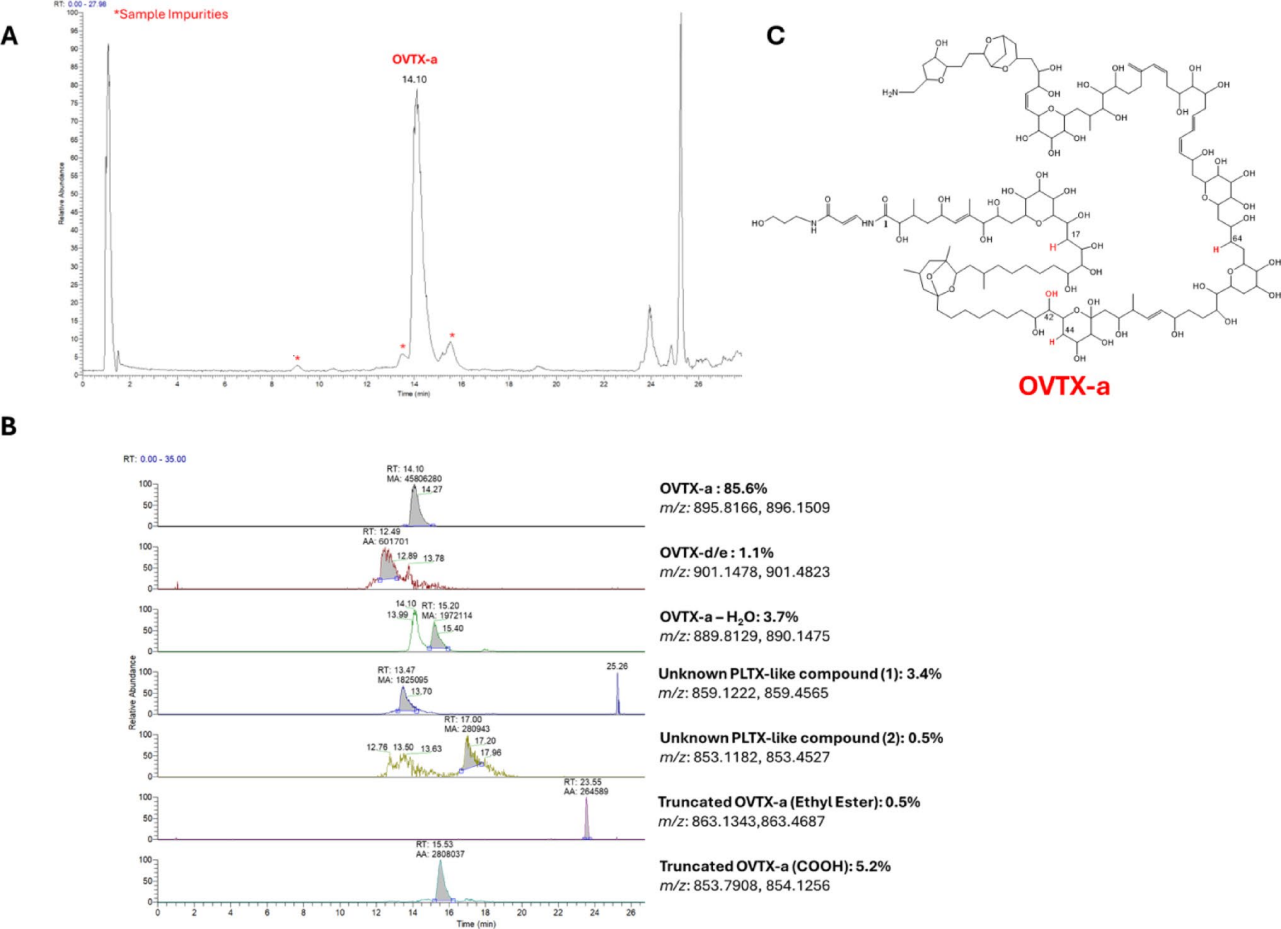
**A** Total Ion Chromatogram (TIC) of the end-product (Sample ID 5a) in EtOH:W (1:1, v/v), slow gradient #2 elution (asterisks indicate sample impurities, while other signals were contained in solvent blank too). **B** XIC of OVTX-a, -d, -e, and other suspect PLTX-like molecules, including OVTX-a degradation products. **C** planar structure of OVTX-a. Truncation of OVTX-a molecule occurs between C1 and N resulting in the formation of a carboxylic acid and an ethyl ester derivative

OVTX-a was purified following the procedure described in Materials and Methods paragraph 2.2. that has been further optimized by Miele et al. (Miele et al. 2024). Quali-quantitative LC-HRMS analysis of the end-product in 50% aqueous ethanol (100 µg OVTX-a /mL), performed shortly after isolation and again after 2 years of storage at − 20 °C, revealed a 74% loss of OVTX-a, with only 25.7 µg/mL remaining. The purity grade of the sample was 86%, as resulted by the LC-HRMS analysis performed in the mass range *m/z* 800–1400 (positive ions). Figure 1 shows the Total Ion Chromatogram (TIC) of the end-product with relevant XIC of the ovatoxins and the degradation products formed throughout the isolation process and storage. Besides the principal component (OVTX-a, 86%), the product contained 1% of OVTX-d/-e and about 13% of degradation products, including de-hydration products of OVTX-a, as well as the carboxylic acid and ethyl ester derivatives of OVTX-a truncated at C1. Noteworthy, the latter degradation product was already observed by Mazzeo et al. (2021) as PLTX methyl ester derivative formed in aqueous methanol solutions under various acidic conditions. Concentration of the end-product was measured by comparing the instrumental response of OVTX-a with that of the PLTX non-certified standard analyzed under the same experimental conditions, assuming that PLTX and OVTX-a present the same molar response (Fig. S3, Table S2).

**Fig. 2:**
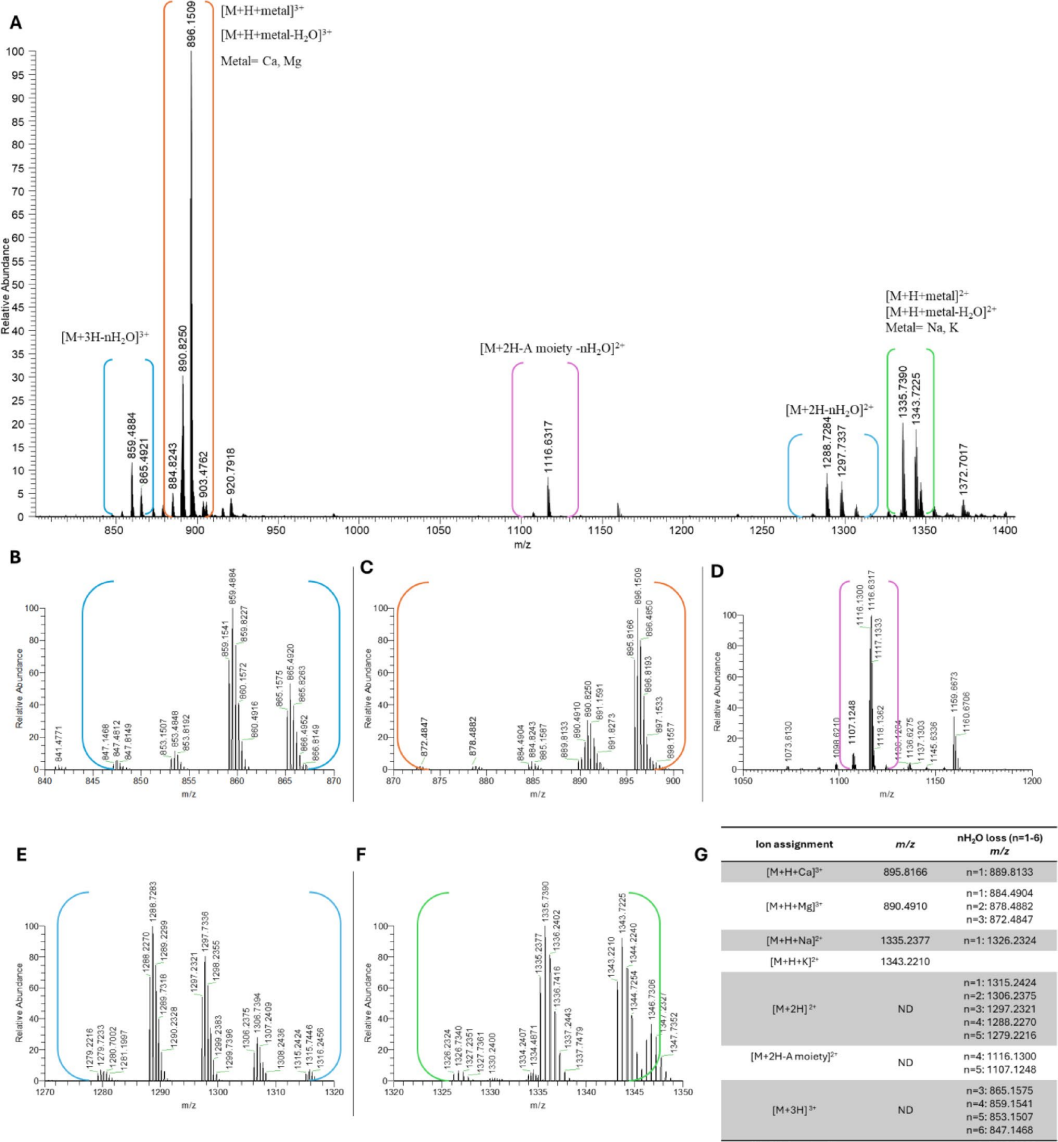
**A** Full scan HRMS of OVTX-a sample ID-5a in the mass range *m/z* 800–1400 with cluster ions assignment in brackets. Enlargements of the region **B** 840–870, **C** 870–900, **D** 1050–1200, **E** 1270–1320, **F** 1320–1350. **G** List of the mono-isotopic ion peaks of each ion cluster and associated water losses. ND = not detected

Figure 2 shows the Full Scan HRMS spectrum of OVTX-a (sample ID 5a) acquired in positive ion mode under slow-gradient #2 conditions. The characteristic ionization behavior of OVTX-a was confirmed as evidenced by the presence of diagnostic triply- and doubly-charged ions in the *m/z* ranges 840–940 and 1050–1400, respectively (Tartaglione et al. 2017) (Fig. 2A). In the triply-charged ion region, the spectrum was dominated by adduct ions formed with Group II metals, namely the [M + H + Ca]^3+^ ion, observed as the base peak, along with the [M + H + Mg]^3+^ ion (Fig. 2C) and a series of in-source fragments corresponding to [M + 3H-nH_2_O]^3+^(Fig. 2B). The doubly-charged ion region displayed the adduct ions formed with Group I metals [M + H + K]^2+^ and the [M + H + Na]^2+^ ions (Fig. 2F), as well as a variety of in-source formed fragments of the type [M + 2H-nH_2_O]^2+^ (Fig. 2E) and [M + 2H-A moiety- nH_2_O]^2+^ (Fig. 2D). Figure 2G reports mono-isotopic ion peaks of each ion cluster and associated water losses.

### Cytotoxicity of OVTX-a

**Fig. 3:**
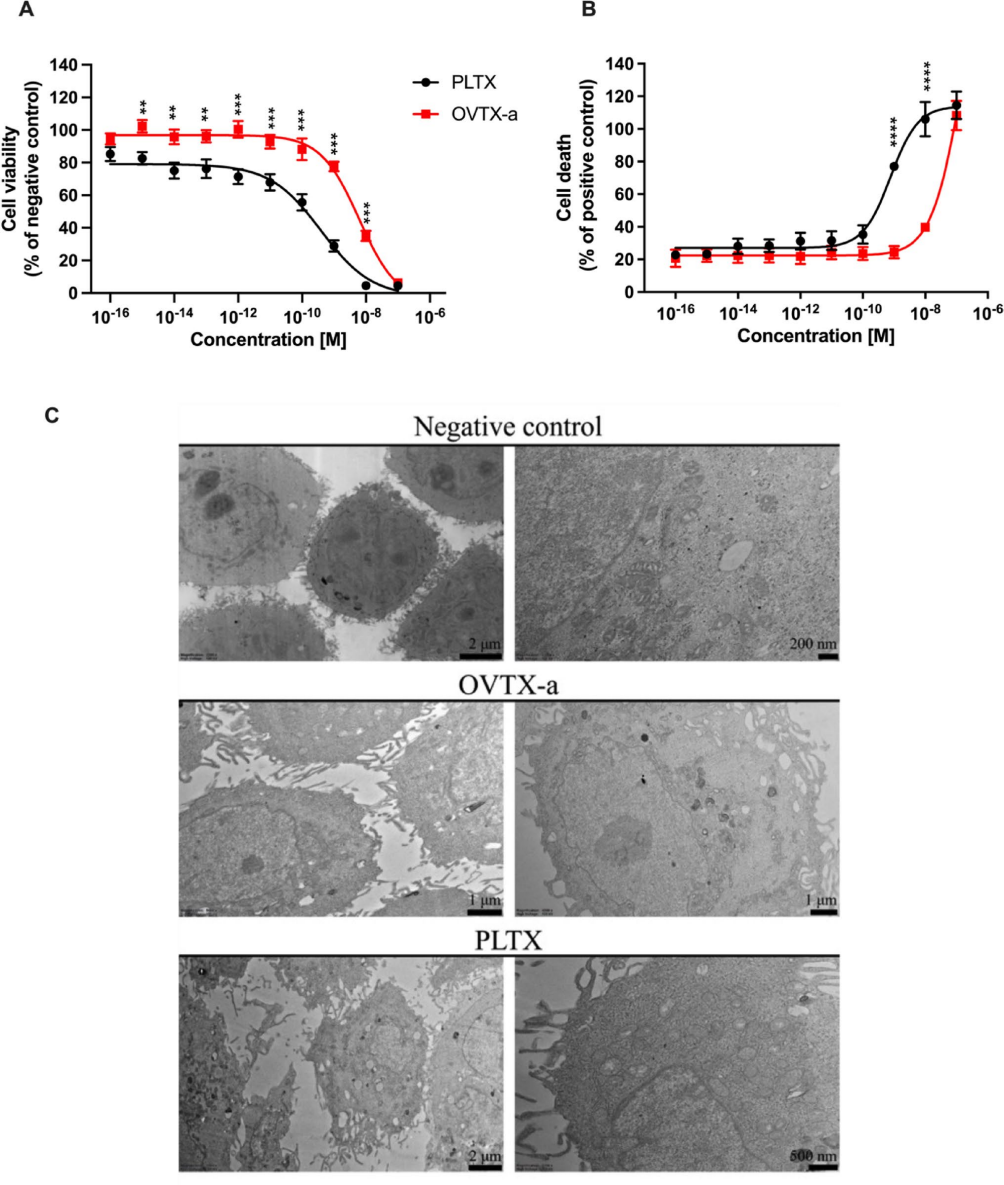
Cytotoxic effects and impact on morphology of OVTX-a on HaCaT cells after 4 h exposure. **A** Effects on cell viability evaluated through the MTT assay; data are reported as % of untreated control. **B** Effects on necrotic-like cell death evaluated measuring LDH cellular release; data are reported as % of positive control (lysed cells). All the results are the means ± SEM of 3 independent experiments performed in triplicate. Significant differences between OVTX-a and PLTX exposed cells: *, *p* < 0.05; **, *p* < 0.01; ****, *p* < 0.0001 (two-way ANOVA and Bonferroni’s post test). **C** Representative TEM images of HaCaT cells after 4 h exposure to OVTX-a or PLTX (1.0 × 10^−9^ M)

The comparative cytotoxic effects of OVTX-a and PLTX (1.0 × 10^−16^–1.0 × 10^−7^ M) were initially evaluated in terms of cell viability reduction and induction of necrotic cell death, after 4 h exposure. Figure 3A shows HaCaT cells viability after exposure to each toxin as compared to negative controls (untreated cells; 100% cell viability), evaluated by the MTT assay. OVTX-a induced a concentration-dependent reduction of cell viability, significant from the concentration of 1.0 × 10^−11^ M (− 7%) up to 1.0 × 10^−7^ M (− 94%), with the concentration reducing cell viability by 50% (EC_50_) equal to 6.1 × 10^−9^ M (95% confidence interval, CI 2.6–14.0 × 10 − ^9^ M). Exposure to PLTX significantly reduced cell viability at the concentration of 1.0 × 10^−14^ M (− 25%), up to 1.0 × 10^–7^ M (− 95%). The EC_50_ of PLTX was 3.8 × 10^−10^ M (95% CI: 1.5–9.8 × 10^−10^ M), more than one order of magnitude lower than that of OVTX-a (*p* < 0.0001). Cell viability reduction induced by OVTX-a was significantly lower than that induced by PLTX within the concentration range of 1.0 × 10^−12^ M (0% and − 29%, respectively; *p* < 0.05) and 1.0 × 10^−8^ M (− 65% and − 95%, respectively; *p* < 0.0001) (Fig. 3A).

Figure 3B shows the effects of each toxin in terms of necrotic-like cell death assessed by LDH cell release. As compared to positive control (100% lysed cells), OVTX-a induced a significant LDH release starting from the concentration of 1.0 × 10^−8^ M (40%) up to 1.0 × 10^–7^ M (109%), which effect was similar to that of the positive control (9% Triton® X-100). PLTX significantly increased LDH release at a concentration of 1.0 × 10^−9^ M (77%) up to 1.0 × 10^−8^ M (105%), similar to that of the positive control. OVTX-a increased LDH release with a significantly lower potency than PLTX at the concentrations of 1.0 × 10^−9^ M (24% and 77%, respectively; *p* < 0.0001) and 1.0 × 10^−8^ M (40% and 106%, respectively; *p* < 0.0001). Overall, these results suggest that OVTX-a cytotoxic potency is lower than that of PLTX.

The effects of OVTX-a and PLTX were also evaluated by TEM analysis of HaCaT cells exposed to each toxin (1.0 × 10^–9^ M) for 4 h. At this concentration, OVTX-a induced a significant but low cell viability reduction, whereas PLTX exerted a higher effect in absence of complete cell death (Fig. 3). As shown by TEM images, negative control cells exhibited a typical rounded and/or polygonal shape with a well-defined plasma membrane and prominent nuclei with visible nucleoli. Moreover, the cytoplasm displays the typical organelles, including copious mitochondria with intact cristae and Golgi apparatus, reflecting active metabolic and protein synthesis processes. On the contrary, visible ultrastructural changes can be noted in toxin-treated keratinocytes. Cells exposed to OVTX-a present visible cytoplasmic vacuoles, moderate elongation and retraction of cytoplasmic processes, shortened and occasionally swollen mitochondria with intact membranes. No clear nuclear alterations, such as chromatin condensation and irregular nuclear envelopes, were observed. On the other hand, PLTX induced dramatic alterations in keratinocytes: pronounced changes in plasma membrane organization, elongation of cytoplasmic processes and abundant cytoplasmic vacuolization, nuclear morphology variations with irregularities of the nuclear envelope as well as swollen mitochondria with disrupted or lost cristae structures indices of compromised mitochondrial integrity and function (Fig. 3C). On the whole, TEM analysis demonstrates ultrastructural alterations in HaCaT cells exposed to OVTX-a, including changes in plasma membrane, mitochondria, nuclei and cellular vacuolization, less pronounced than those provoked by PLTX exposure.

### Mitochondrial-related cellular alterations induced by OVTX-a

**Fig. 4:**
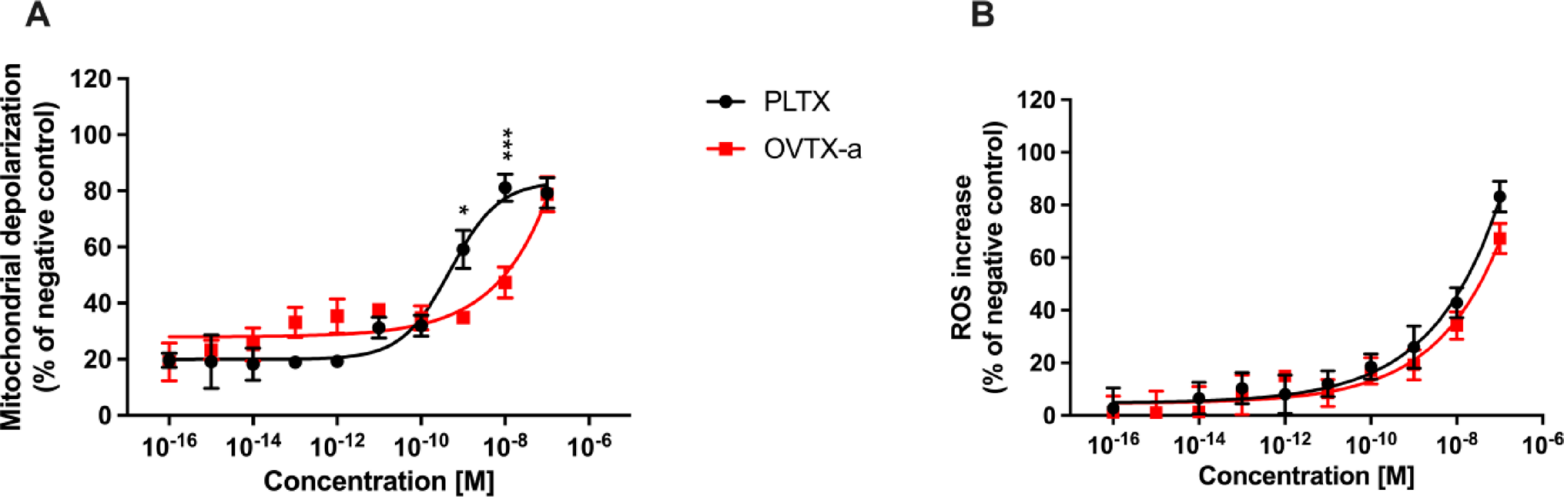
Mitochondrial related disfunctions in HaCaT cells exposed to OVTX-a or PLTX. **A** Mitochondrial depolarization evaluated by the JC-1 assay after 4 h exposure. **B** ROS production evaluated by the NBT reduction assay after 1 h exposure. Results are reported as % of negative control and are the means ± SEM of 3 independent experiments performed in triplicate. Significant differences between OVTX-a and PLTX exposed cells: *, *p* < 0.05; ***, *p* < 0.001 (two-way ANOVA and Bonferroni’s post-test)

To investigate the role of mitochondrial dysfunction in OVTX-a-induced cytotoxicity, mitochondrial depolarization (JC-1 assay) and ROS production (NBT reduction assay) were evaluated in treated cells (Fig. 4). Compared to negative controls, 4 h exposure of HaCaT cells to OVTX-a (1.0 × 10^−16^–1.0 × 10^−7^ M) induced a significant increase of mitochondrial depolarization from the concentration of 1.0 × 10^−11^ M (38%) up to 1.0 × 10^−7^ M (79%). Similarly, also PLTX significantly increased mitochondrial depolarization from the concentration of 1.0 × 10^–11^ M (31%) up to 1.0 × 10^−7^ M (79%). However, the effect of OVTX-a was significantly lower than that of PLTX at the concentrations of 1.0 × 10^−9^ M (35% and 59%, respectively; *p* < 0.05) and 1.0 × 10^−8^ M (47% and 81%, respectively; *p* < 0.001). As positive control, valinomycin (0.1 µg/mL) induced 74% increase of mitochondrial depolarization (Fig. 4A).

The ability of OVTX-a to induce oxidative stress was assessed by the NBT reduction assay, that measures the intracellular level of superoxide anion. One hour exposure to OVTX-a (1.0 × 10^−16^–1.0 × 10^−7^ M) induced a concentration-dependent increase of superoxide anion, comparable to that induced by PLTX, starting from the concentration of 1.0 × 10^−10^ M (17% and 19%, respectively; *p* > 0.05) up to 1.0 × 10^−7^ M (67% and 83%, respectively; *p* > 0.05). As positive control, AAPH (1.0 × 10^−2^ M) increased ROS production by 137% (Fig. 4B).

Overall, these results indicate that OVTX-a induces mitochondrial depolarization with a potency lower than that of PLTX, but both the toxins induce a comparable increase of ROS production.

### Assessment of OVTX-a mode-of-action

OVTX-a mode-of-action was characterized considering the molecular target of PLTX, i.e. the Na^+^/K^+^ ATPase, and the known mechanisms involved in PLTX effects towards skin keratinocytes, i.e. the involvement of flavonic-based oxidative enzymes in the ROS-dependent cytotoxicity. Initially, to verify if OVTX-a shares the same molecular target of PLTX, the toxins’ effects were evaluated in presence of OUA, a blocker of the Na^+^/K^+^ ATPase: cells were pre-exposed to OUA (1.0 × 10^−5^ M) for 1 h and then co-exposed to each toxin (1.0 × 10^−11^–1.0 × 10^−7^ M) for 4 h (assessment of cell viability reduction, necrotic-like cell death and mitochondrial depolarization) or 1 h (assessment of ROS production).

**Fig. 5:**
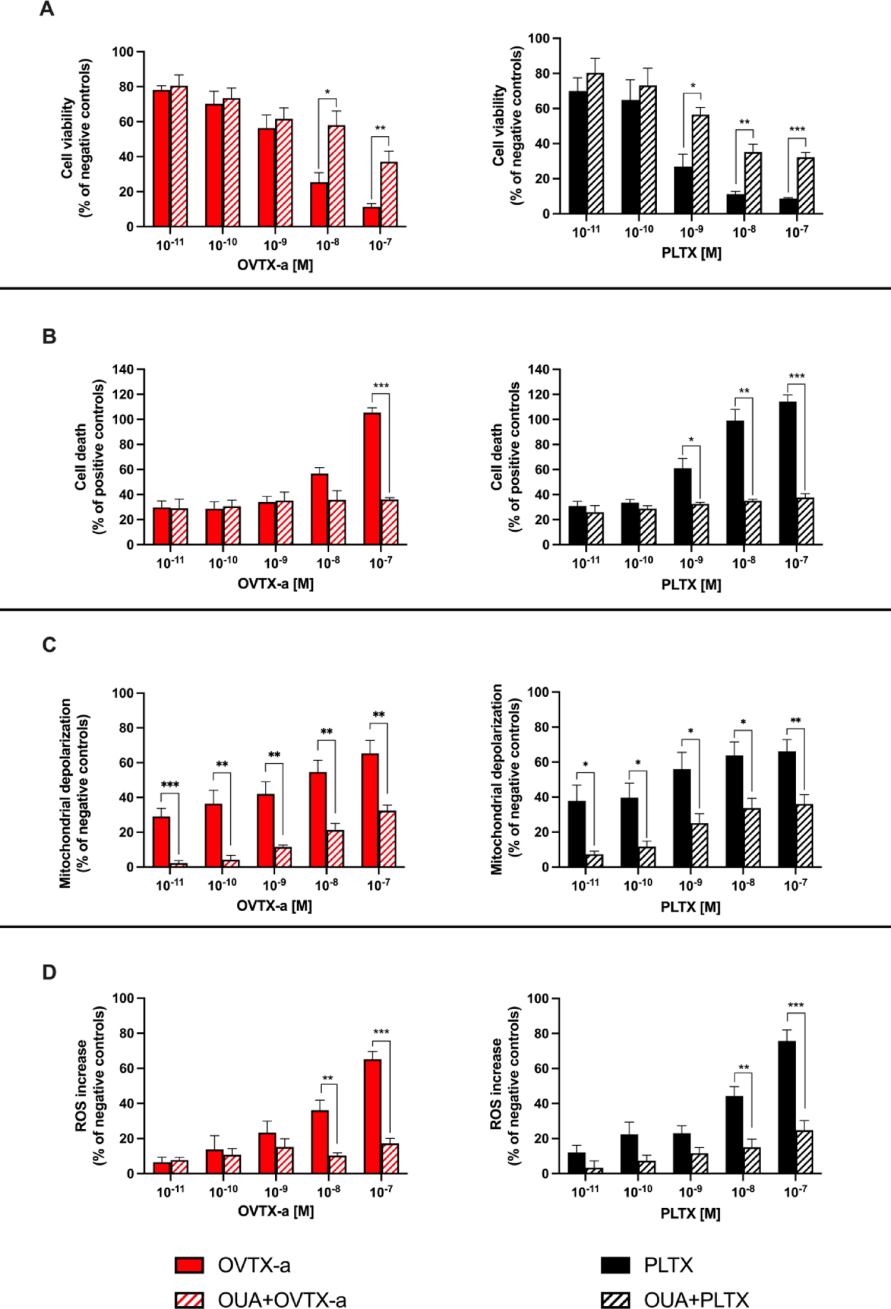
Inhibitory effect of ouabain (OUA) on the cytotoxic effects of OVTX-a and PLTX. Cytotoxicity was measured in terms of **A** cell viability by MTT assay, **B** necrotic-like cell death by LDH release assay, **C** mitochondrial depolarization by JC-1 assay and **D** ROS production by NBT assay. Cells were pre-exposed to 1.0 × 10^−5^ M OUA for 1 h and subsequently co-exposed to OVTX-a or PLTX for 4 h or 1 h for the NBT assay. In parallel, cells were exposed to each toxin alone, without pre-exposure to OUA. Data are expressed as % of negative or positive control and are the mean ± SEM of 3 independent experiments performed in triplicate. Significant differences between cells exposed to OVTX-a or PLTX alone and cells co-exposed to each toxin and OUA: *p < 0.05; **p < 0.01; ***p < 0.001 (Student’s t-test)

Figure 5 shows the effects of OVTX-a or PLTX with or without OUA in terms of cell viability reduction (MTT assay), necrotic cell death (LDH release assay), mitochondrial membrane depolarization (JC-1 assay) and ROS production (NBT assay). Viability of cells exposed only to 1.0 × 10^–8^ M or 1.0 × 10^−7^ M OVTX-a (25% or 11%, respectively) was significantly increased by OUA at 58% (*p* < 0.05) and 37% (*p* < 0.01), respectively. The effect of OUA on PLTX-induced reduction in cell viability was evident already at the concentration of 1.0 × 10^−9^ M: viability of cells exposed only to 1.0 × 10^−9^ M PLTX (27%) was significantly increased by OUA at 56% (*p* < 0.05), whereas cells viability after exposure to the highest PLTX concentration (10^−7^ M, 9%) was increased by OUA at 32% (*p* < 0.001) (Fig. 5A).

OUA was able to reduce the necrotic-like cell death induced by OVTX-a or PLTX, assessed as LDH release. As compared to positive control, LDH release induced by 1.0 × 10^−7^ M OVTX-a (105%) was significantly reduced by OUA at 36% (*p* < 0.001). On the other hand, LDH release induced by PLTX at the concentration ranging from 1.0 × 10^−9^ M (61%) to 10^−7^ M (114%) was reduced by OUA at levels between 26% (*p* < 0.05) and 38% (*p* < 0.001), respectively (Fig. 5B).

OUA inhibited also the effects of both toxins on mitochondrial depolarization (Fig. 5C) in a similar manner. In particular, mitochondrial depolarization induced by OVTX-a concentrations ranging from 1.0 × 10^−11^ M (29%) to 1.0 × 10^−7^ M (65%) was reduced by OUA at levels ranging from 2% (*p* < 0.001) to 32% (*p* < 0.01), respectively. Similarly, mitochondrial membrane depolarization induced by PLTX concentrations ranging from 1.0 × 10^−11^ M (38%) to 1.0 × 10^−7^ M (66%) was reduced by OUA at levels ranging from 7% (*p* < 0.05) to 36% (*p* < 0.01), respectively (Fig. 5C).

Also, the ROS production increase associated with each toxin was significantly reduced by cells’ co-exposure to OUA in a similar manner. ROS production increased by OVTX-a concentrations of 1.0 × 10^−8^ M (36%) and 1.0 × 10^–7^ M (65%) was significantly reduced by OUA at 10% (*p* < 0.01) and 17% (*p* < 0.001), respectively. As reference, ROS production increased by PLTX concentrations of 1.0 × 10^−8^ M (39%) and 1.0 × 10^−7^ M (76%) was reduced by OUA at 15% (*p* < 0.01) and 25% (*p* < 0.001), respectively (Fig. 3D).

Overall, these results suggest that, notwithstanding the cellular event, OUA significantly reduces the cytotoxic effects of both OVTX-a and PLTX, suggesting that these toxins share the same molecular target, i.e. Na^+^/K^+^ ATPase.

Subsequently, the mechanisms involved in the oxidative cytotoxic effects were investigated by co-exposing the cells to DPI, an inhibitor of flavonic-based oxidative enzymes, such as NADPH oxidase (NOX) and nitric oxide synthase (NOS), playing a significant role in PLTX-induced ROS production in skin keratinocytes. Cells were pre-exposed to DPI (5.0 × 10^–6^ M) for 1 h and subsequently co-exposed to each toxin (1.0 × 10^−11^– 1.0 × 10^−7^ M) for 4 h or 1 h as previously described for OUA.

**Fig. 6:**
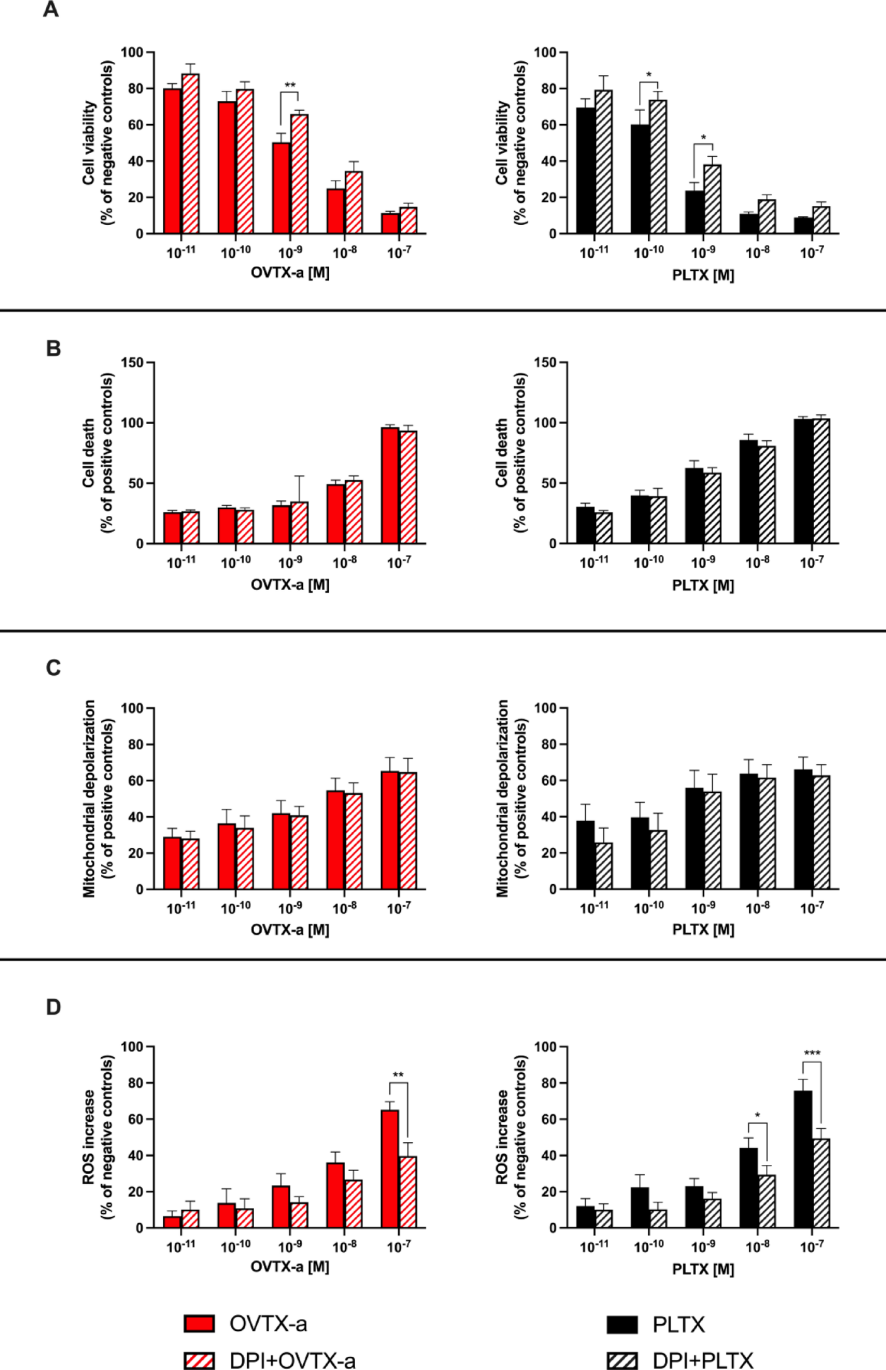
Inhibitory effect of diphenyleneiodonium chloride (DPI) on the cytotoxic effects of OVTX-a and PLTX. Cytotoxicity was measured in terms of **A** cell viability by MTT assay, **B** necrotic-like cell death by LDH release assay, **C** mitochondrial depolarization by JC-1assay and **D** ROS production by NBT assay. Cells were pre-exposed to 5.0 × 10^−6^ M DPI for 1 h and subsequently co-exposed to OVTX-a or PLTX for 4 h or 1 h for the NBT-assay. In parallel, cells were exposed to each toxin alone, without pre-exposure to DPI. Data are expressed as % of negative or positive control and are the mean ± SEM of 3 independent experiments performed in triplicate. Significant differences between cells exposed to OVTX-a or PLTX alone and cells co-exposed to each toxin and DPI: *p < 0.05; **p < 0.01; ***p < 0.001 (Student’s t-test)

Viability of cells exposed to 1.0 × 10^−9^ M OVTX-a alone (50%) was slightly but significantly increased by co-exposure to DPI at 66% (*p* < 0.01). For PLTX, the effect of DPI was significant at the toxin concentrations of 1.0 × 10^−10^ M and 1.0 × 10^−9^ M: these concentrations reduced cells viability at 60% and 24%, which were increased by DPI at 74% and 38% (*p* < 0.05), respectively (Fig. 6A).

With regard to necrotic-like cell death, DPI did not significantly counteract toxin-induced plasma membrane damage, since no reduction was observed in LDH release by cells exposed to any concentration of OVTX-a or PLTX (Fig. 6B). Similarly, DPI did not significantly affect OVTX-a or PLTX-induced mitochondrial depolarization (Fig. 6C).

In contrast, ROS production in cells exposed to high concentrations of OVTX-a or PLTX was reduced by cells co-exposure to DPI. In particular, ROS production induced by 1.0 × 10^−7^ M OVTX-a (65%) was significantly reduced by DPI at 40% (*p* < 0.01). ROS production increased by PLTX at the concentrations of 1.0 × 10^−8^ M (44%) and 1.0 × 10^−7^ M (76%) was significantly reduced by DPI at levels of 29% (*p* < 0.05) and 49% (*p* < 0.001), respectively (Fig. 6D).

## Discussion

OVTXs are polyether marine toxins, structurally related to PLTX, identified in *Ostreopsis* spp(Gu et al. 2022). These dinoflagellates bloom frequently in temperate and tropical waters, causing respiratory and skin symptoms and signs in humans exposed to marine aerosols or seawater (Gallitelli et al. 2005; Brescianini et al. 2006; Ciminiello et al. 2006; Durando et al. 2007; Kermarec et al. 2008; Tichadou et al. 2010; Tubaro et al. 2011). In case of skin exposure, adverse effects include dermatitis, itching, erythema and rashes (Tubaro et al. 2011). Subsequent studies (Tartaglione et al. 2016) indicated that the causative agent of these effects was OVTX-a, identified as the primary toxin produced by the Mediterranean strains of *O.* cf. *ovata* (Ciminiello et al. 2006, 2008) and *O. fattorussoi* (Accoroni et al. 2016).

Hence, this in vitro study was carried out on human HaCaT keratinocytes, that already demonstrated to be sensitive to PLTX effects (Pelin et al. 2011) as well as a good model for assessing skin toxicity of dermotoxic compounds (Boukamp et al. 1988; Gibbs 2009). A comparative approach was followed to assess differences in cytotoxic potential and the mode-of-action between OVTX-a and PLTX. In particular, OVTX-a cytotoxic effects on HaCaT cells were investigated in a wide range of concentrations (1.0 × 10^−16^–1.0 × 10^−7^ M) up to 4 h exposure, by evaluating cellular parameters known to be affected by PLTX in the same cellular model: cell viability reduction (MTT assay), necrotic-like cell death (LDH release), mitochondrial depolarization (JC-1 assay) and ROS production (NBT assay) (Pelin et al. 2011, 2013a, b, 2014, 2016a). Our results show significant differences between OVTX-a and PLTX cytotoxic potencies for most of these parameters, except for ROS production. In fact, OVTX-a reduced HaCaT cell viability with an EC_50_ of 6.1 × 10^−9^ M, being more than one order of magnitude less toxic than PLTX (EC_50_ = 3.8 × 10^−10^ M). In a similar manner, OVTX-a induced a necrotic-like cell death with a significantly lower potency compared to PLTX. Moreover, ultrastructural alterations in cells exposed to OVTX-a recorded by TEM (moderate elongation and retraction of cytoplasmic processes, formation of some cytoplasmic vacuoles, shortened and occasionally swollen mitochondria) were less pronounced than those recorded in cells exposed to PLTX (plasma membrane and nuclear alterations, high cellular vacuolization and severe mitochondrial damages visible as swollen mitochondria with disrupted or lost cristae). Given these findings and considering previous observations on PLTX sequential effects in HaCaT keratinocytes involving early mitochondrial damage followed by oxidative stress (Pelin et al. 2011, 2013b, 2014), the cytotoxic effects of OVTX-a were also evaluated in terms of mitochondrial depolarization and ROS production. Our results demonstrate that OVTX-a led to a concentration-dependent increase of both cellular parameters. Compared to PLTX, mitochondrial depolarization induced by OVTX-a was significantly lower, but the two toxins induced a comparable increase of ROS production. The latter result could be ascribed to a lower sensitivity of the NBT assay compared to the assays evaluating other parameters used in the study. In any case, overall, the results suggest that OVTX-a has a lower cytotoxic potency towards HaCaT cells than the reference compound PLTX. However, it should be noted that cytotoxicity of OVTX-a falls in the nanomolar range, far lower than that of other compounds of anthropogenic origin that are well known to be toxic by skin contact, such as polycyclic aromatic hydrocarbons (i.e. benzene, toluene), which exert their cytotoxicity in keratinocytes at millimolar concentrations (Bahri et al. 2010) or bisphenol A, (Kim et al. 2024) exerting cytotoxicity at micromolar/sub-millimolar concentrations. Thus, OVTX-a is a thousand to a million times more toxic on keratinocytes than mutagenic compounds, such as polycyclic aromatic hydrocarbons, and other skin sensitizing agents reported in the European Chemical Agency database, ([CSL STYLE ERROR: reference with no printed form.]) such as bisphenol-A.

The cytotoxicity observed for OVTX-a (EC_50_ = 6.1 × 10^−9^ M) in this study aligns with preliminary results obtained earlier using the same cell model (EC_50_ = 1.1 × 10^−9^ M) (Pelin et al. 2016b) substantiating that, in keratinocytes, OVTX-a exhibits a lower cytotoxic potential than PLTX. A reasons for this difference could be ascribed to the presence of a hydroxyl group in position C44 of PLTX, that is lacking in OVTX-a molecule and that, besides stereochemistry, appears to play a significant role in the cytotoxic activity of this toxin (Pelin et al. 2016b; Melchiorre et al. 2025). The about fivefold difference in EC_50_ of OVTX-a measured between this and the earlier study (Pelin et al. 2016b) could be related to the different purity degree of OVTX-a samples (86% versus 51%). An about 20-fold difference was, instead, observed between EC_50_ of PLTX measured in this study (3.8 × 10^–10^ M) and that measured earlier (1.8 × 10^−11^ M) (Pelin et al. 2016b), which is most likely related to variability in composition of commercially-available PLTX lots.

The results of the present study contrast with those reported in a recent in vitro study on different cell lines, showing a similar potency between OVTX-a and PLTX towards HaCaT keratinocytes, either after 4 h or 24 h exposure (Lanceleur et al. 2024). After 4 h, the authors recorded an OVTX-a IC_50_ of 2.3 × 10^−9^ M, only slightly lower than that of PLTX (2.9 × 10^−9^ M) (Lanceleur et al. 2024). It should be underlined that OVTX-a IC_50_ value of 2.3 × 10^−9^ M reported by Lanceleur et al. (2024)(Lanceleur et al. 2024) is slightly lower than that measured herein (6.1 × 10^−9^ M). It is of note that there could be structural differences between the OVTX-a samples tested in the two studies: OVTX-a used by Lanceleur et al. (2024) was isolated from Mediterranean strains of *O.* cf. *ovata* (MCCV54 and MCCV55), its purity grade was not specified, and its LC-HRMS data (Full Scan HRMS and MS^n^ spectra, chromatograms, etc.) or other spectroscopic data are not reported. Given the lack of a complete characterization, it is not possible to ascertain whether the tested molecule was the same molecule and/or had a similar purity as the one tested herein and thus a direct comparison between toxicological data is not possible.

Furthermore, a high difference of about 1 order of magnitude can be noticed between IC_50_ of PLTX reported by Lanceleur (2024) after 4 h exposure (2.9 × 10^−9^ M) and that reported herein (3.8 × 10^−10^ M). This discrepancy could be again due to differences in composition of PLTX lots and/or to the solvent used for toxin solubilization, namely DMSO in Lanceleur et al. (2024) and 50% aqueous EtOH in our study. Even if both studies considered the solvent effect by using control samples, it cannot be excluded that 2% DMSO and 50% aqueous EtOH have a different effect on toxin bioavailability that in turn results in different cytotoxic potency. In addition, a 2% DMSO concentration is known to be cytotoxic, thus possibly masking PLTX cytotoxicity (Ilieva et al. 2021; Strus et al. 2024). Ultimately, it is worth noting that PLTX and OVTX-a used by Lanceleur et al. (2024) were quantified by LC-HRMS in MeOH-DMSO 8:2 (v/v) while those used in this study were quantified by LC-HRMS in 50% aqueous EtOH. The LC–MS response of PLTX-like compounds in solution has been demonstrated to be different depending on solvent (Mazzeo et al. 2021; Melchiorre et al. 2025) which in turn may affect quantitation.

In the second part of the study, we assessed whether OVTX-a could share the same mode-of-action with PLTX, despite its lower cytotoxic potency. It is widely known that PLTX binds to the Na^+^/K^+^ ATPase, converting it into a non-selective cation channel leading to a cellular ionic imbalance (Habermann 1989; Hammond and Roy 2024). Hence, OVTX-a mode-of-action was characterized in reference to that of PLTX, focusing on two major aspects: (i) the involvement of the Na^+^/K^+^ ATPase as molecular target and (ii) the involvement of flavonic-based oxidative enzymes in the mechanism of ROS-dependent cytotoxicity. For the first case, OVTX-a and PLTX cytotoxic effects were evaluated in presence of OUA, a well-known blocker of the Na^+^/K^+^ ATPase, whose effectiveness in inhibiting in vitro PLTX effects was already demonstrated (Habermann and Chhatwal 1982; Ledreux et al. 2009; Pelin et al. 2013a). OUA significantly inhibited the cytotoxic effects of OVTX-a assessed by different parameters indices of cell viability, necrotic-like death, mitochondrial depolarization and ROS. Similarly, OUA significantly reduced the cytotoxic effects of PLTX, albeit with a higher potency. These results suggest that OVTX-a and PLTX share the same molecular target, the Na^+^/K^+^ ATPase, corroborating our previous findings on the ability of OVTX-a to bind the Na^+^/K^+^ ATPase on keratinocytes (Pelin et al. 2016b). Subsequently, to investigate whether OVTX-a shares the mechanisms involved in oxidative cytotoxic damage with PLTX, the cytotoxic effects of each toxin were evaluated in presence of DPI, an inhibitor of flavoprotein-based enzymes, such as NOX and NOS, known to be involved in PLTX-induced oxidative stress in keratinocytes (Pelin et al. 2013b). DPI significantly reduced the cell viability decrease and ROS production induced by both OVTX-a and PLTX, whereas no inhibitory effects were observed for mitochondrial depolarization and necrotic-like cell death. This result is in line with previous findings in HaCaT keratinocytes exposed to PLTX, where ROS production contributes to the reduced cell viability by the toxin, occurring as a secondary effect following early mitochondrial dysfunction in the mechanism of PLTX cytotoxicity (Pelin et al. 2013b). In any case, these results suggest that OVTX-a and PLTX share not only the same molecular target, but also the same mode-of action, involving NOX and NOS activation in ROS-dependent reduction of cell viability, as previously demonstrated for PLTX (Pelin et al. 2013b).

In conclusion, this study provides a significant contribution to the characterization of the toxic effects of OVTX-a in human skin keratinocytes and, for the first time, elucidates its mechanisms of action. Specifically, the highly purified OVTX-a appeared to be less cytotoxic on HaCaT cells than the reference compound PLTX, although it shares the same molecular target and mode-of-action. Notwithstanding, OVTX-a exerts significant cytotoxic effects at nanomolar concentrations after exposure times as short as 4 h, highlighting its potential hazard at the skin level. At an environmental level, it should be noted that most of the analyzed *O.* cf. *ovata* strains from the Mediterranean area present an overall OVTX content in the range 4–238 pg/cell (Tartaglione et al. 2017). With regards to only OVTX-a (C_129_H_223_N_3_O_52_, theoretical mass 2646.4892), the toxin content spans from 6 to 118 pg/cell, namely 2.3 to 45 fmol/cell. When an *O.* cf. *ovata* bloom reaches 1 × 10^6^ cell/L, an OVTX-a concentration of 2.3–45 nM is expected. Thus, considering the EC_50_ of 6.1 nM found in the present study, it is feasible that OVTX-a might exert toxic effects on skin cells. The results obtained are consistent with, and could mechanistically explain, the cutaneous effects experienced by bathers during *O.* cf. *ovata* blooms, based solely on OVTX-a, although the effects of other ovatoxins remain largely unexplored to date.

## Supplementary Information

Below is the link to the electronic supplementary material.

Supplementary Material 1
