# Supplementary material for: Cutaneous effects of ovatoxin-a: an in vitro study on human skin keratinocytes
Source: Arch Toxicol. 2025 Oct 31;100(2):773–88. doi: 10.1007/s00204-025-04229-3 (PMC12886334; doi:10.1007/s00204-025-04229-3)
Supplement: Supplementary file 1 — Supplementary Material 1 [file 204_2025_4229_MOESM1_ESM.pdf]

Cutaneous effects of ovatoxin-a: *in vitro* study on human skin keratinocytes

Alessandra D'Arelli[1], Michela Carlin[1], Silvio Sosa[1], Chiara Melchiorre[2,3], Fabio Varriale[2], Luciana Tartaglione[2], Michela Varra[2], David Kulis[4], Donald M. Anderson[4], Mark Poli[5], Aurelia Tubaro[1], Carmela Dell'Aversano[2], Marco Pelin[1]

[1]University of Trieste, Department of Life Sciences, Trieste, Italy

[2]University of Naples Federico II, School of Medicine and Surgery, Department of Pharmacy, Naples, Italy, Via Domenico Montesano 49, 80131, Naples, Italy.

[3]NBFC, National Biodiversity Future Center, Palermo, Italy.

[4]WHOI, Woods Hole Oceanographic Institution, Woods Hole, MA 02543 USA

[5]USAMRIID, U.S. Army Medical Research Institute of Infectious Diseases, Diagnostic Systems Division, Ft Detrick, MD, USA

Method S1. *Ostreopsis* cf. *ovata* culture as starting material

17 L batch cultures of NIES3351 were grown in triplicate 20 L pyrex carboys for toxin production. For this, autoclaved carboys were filled with 17 L of sterile filtered Vineyard Sound seawater using a 0.2 µm, Whatman Polycap 75 capsule filter followed by nutrient enrichment and inoculation with 1 L of exponentially growing culture. These carboys were constantly bubbled with purified, sterile air to help minimize CO_2 depletion and pH change as well as to help keep the cells resuspended during high-density growth. Following approximately 4 weeks of incubation, the cultures were harvested in a laboratory fume hood by pressurizing the carboy with air forcing the contents through a sampling port into a 10 µm Nitex net. Captured cells from each carboy were then rinsed into four 250 mL conical centrifuge bottles which were spun at 1000 x g for 10 min at 23 °C. The resulting 4 cell pellets were combined into a pre-weighed 50 mL conical centrifuge tube. The 50 mL tubes were centrifuged as above, the supernatant removed, and the tubes weighed again to determine the wet weight of algae collected from each carboy. The cell pellets were stored frozen at -80°C and shipped on dry ice to the University of Napoli Federico II for toxin extraction and purification.

Method S2. OVTX-a extraction, purification and quantitation

Isolation of ovatoxin-a was carried out by a 4-step procedure, including Extraction, Medium Pressure Liquid Chromatography (MPLC), and two preparative High Performance Liquid Chromatography (HPLC) purification steps. Briefly, the algal pellet obtained from 51 L of *O.* cf *ovata* NIES3351 culture containing approximately 8×10^6 cells/L was added to 11.6 L of MeOH:W (1:1, v/v). A ratio of ~30 mL of extracting solvent per million cells was used. The suspension was split in 50 mL PP tubes and sonicated at 20% amplitude for 10 minutes in pulse mode (1-second stop-time) in an ice bath by means of Bandelin UW 2200 equipped with Bandelin Sonoplus probe (BANDELIN electronic GmbH & co. KG, Berlin, Germany). The aliquots were centrifuged at 9000 rpm for 10 minutes at room temperature, the supernatants were pooled and concentrated to 100 mL by means of rotavapor HEIDOLPH HEY-VAP Core HL (Heidolph Instruments GmbH & Co. KG, Schwabach, Germany) equipped with VACUUBRAND™ VARIO™ Chemistry Pumping Unit, PC 3001 (VACUUBRAND GMBH + CO KG, Wertheim, Germany). A 20 µL- aliquot of the crude extract was diluted 1:10, 1:100 and 1:1000 with MeOH:W (1:1, v/v) and analyzed by LC-HRMS to identify the toxin profile. The concentrated crude extract underwent MPLC clean-up by means of a Combiflash® Rf 200 (Teledyne Isco, Nebraska, USA) equipped with an UV detector (λ233 and 263 nm). Briefly two 50 mL-aliquots of the

crude extract were loaded on RediSep ® Rf C18 360g, 40-63 µm, 60 Å, mesh 230-400, column volume 361.1 mL (Teledyne Isco) previously equilibrated with 2 column volumes (CV) of W (A) 60% and 2-PrOH (B) 40% at a flow rate of 7.0 mL/min (2 CV=720mL). Ladder-like gradient: time (t)= 0–10 min 40%B (0.2 CV=72 mL); t= 10–25 min 70%B (0.3 CV=108 mL); t= 25–50 min 90%B (2 CV=720 mL); t= 50–75 min 95%B (2 CV=720 mL); t= 75–85 min 90%B (0.7 CV=252 mL).The flow rate was 7 mL/min from t= 0 to 25 min, then increased to 25 mL/min from t= 25 to 85 min. Noteworthy, both the extraction and the MPLC-clean-up steps were recently further optimized by Miele et. al (2024).

The MPLC fractions richest in OVTXs were pooled (2.6 L) and concentrated under N2 stream at room temperature by a Labortechnik TM-130-36 Nitrogen evaporator (Liebisch GmbH & Co, Bielefeld, Germany), with ACN added during the concentration process up to a final volume of 2 mL ACN:W 2:8 (v/v). This fraction underwent HPLC-step 1 under the same experimental conditions reported by Miele et al. (2024). The semi-purified fraction (300 mL), containing all the OVTXs was concentrated at room temperature under N2 stream to 2.0 mL and then subjected to HPLC-step 2, to separate OVTX-a from the other congeners. HPLC-step 2 was accomplished under the same experimental conditions optimized by Miele et al. (2024)36. A 3 mL-aliquot of the whole purified fraction (50 mL), containing approximately 200 µg of pure OVTX-a was freeze-dried, reconstituted in 1 mL of EtOH:W (1:1, v/v), and re-quantified (187 µg) by LC-HRMS as described below. The sample product was diluted with EtOH:W (1:1, v/v) to a final concentration of 100 µg/mL and stored at -20°C for two years (sample ID-5a). Re-quantification and purity assessment was performed by LC-HRMS just before shipment to the University of Trieste to assess whether any degradation of OVTX-a occurred over the 2-year storage period.

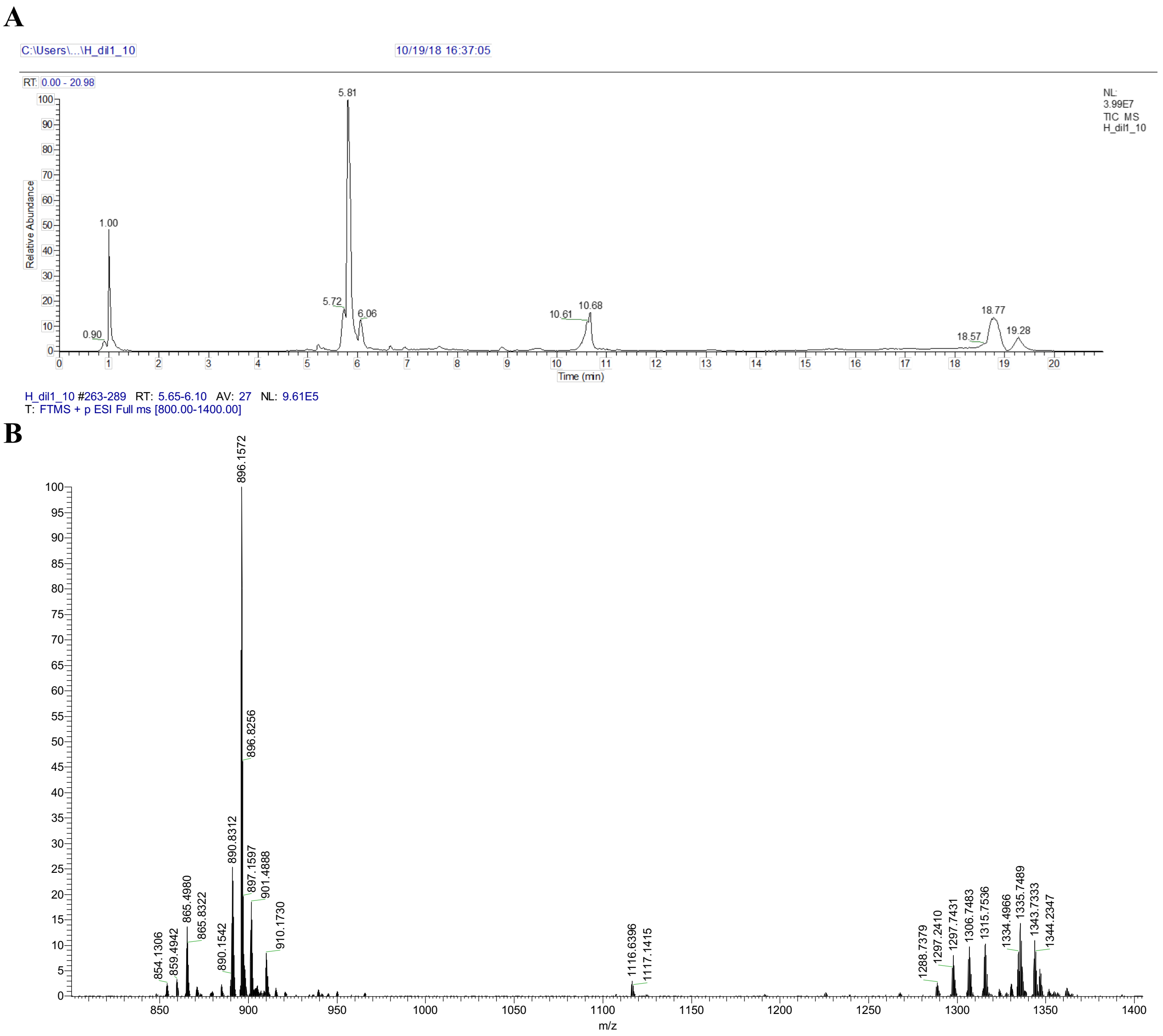

Fig. S1 (A) Total Ion Chromatogram (TIC) of the *O.* cf. *ovata* (NIES 3351) crude extract diluted 1:10 in MeOH:W, (1:1 v/v), and (B) Full scan HRMS spectrum (*m*/*z* 800-1400) of the chromatographic peaks eluting in the range 5.65-6.10 min, under fast gradient elution (Miele et al. 2024).

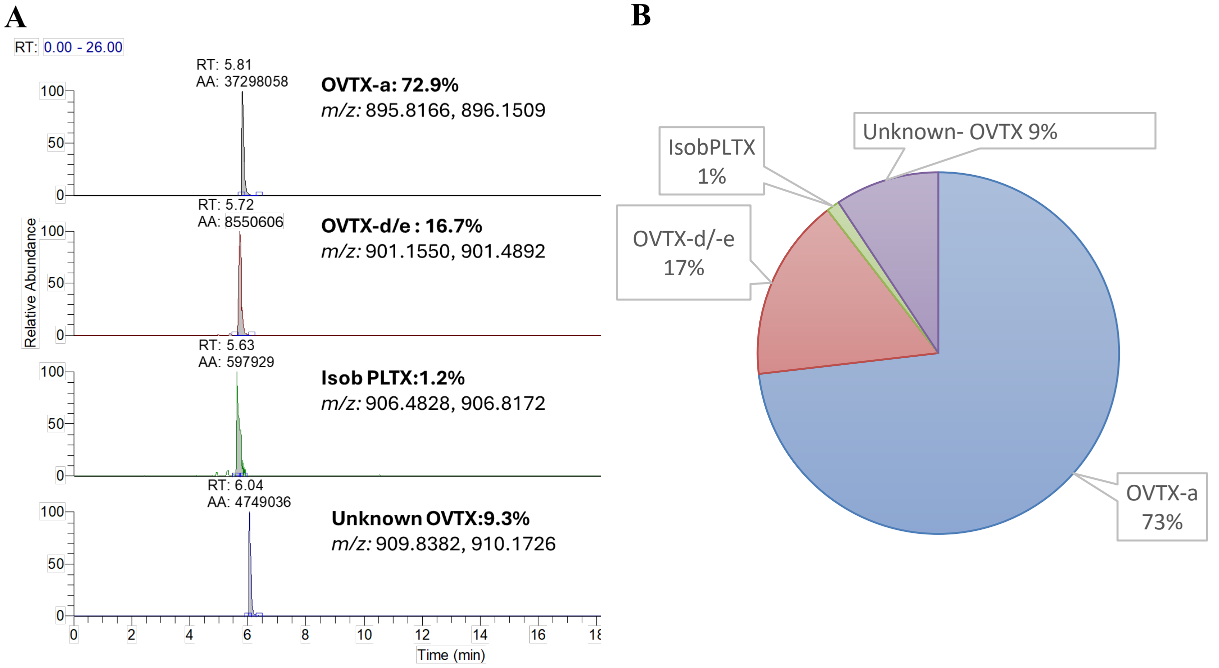

Fig. S2 (A) Extracted ion chromatograms obtained by selecting the exact mass of the mono-isotopic ion and the most intense ion peaks of the $[M+H+Ca]^{3+}$ ion cluster of each OVTX congener from total ion current of the Full-scan HRMS experiment and (B) relative percentages of the ovatoxins contained in the *O.* cf. *ovata* (NIES 3351) crude extract diluted 1:10 in MeOH:W(1:1, v/v).

Table S1 Elemental formulas, theoretical mono-isotopic and most abundant ion peaks of the $[M+H+Ca]^{3+}$ ion cluster of ovatoxins (OVTXs) and degradation products (Miele et al., 2024) searched in the full scan HRMS spectrum of OVTX-a (Sample ID 5a) to measure its purity grade. In bold are highlighted the OVTX-a and relative congeners/degradation products found in the end-product (sample ID 5a).

Name	Acronym	Formula	Mono-isotopic peak $[M+H+Ca]^{3+}$	Most abundant peak $[M+H+Ca]^{3+}$
**Ovatoxin-a**	**OVTX-a**	$C_{129}H_{223}O_{52}N_3$	**895.8195**	**896.1540**
Ovatoxin-b	OVTX-b	$C_{131}H_{227}O_{53}N_3$	910.4949	910.8294
Ovatoxin-c	OVTX-c	$C_{131}H_{227}O_{54}N_3$	915.8266	916.1610
**Ovatoxin-d**	**OVTX-d**	$C_{129}H_{223}O_{53}N_3$	**901.1511**	**901.4856**
**Ovatoxin-e**	**OVTX-e**	$C_{129}H_{223}O_{53}N_3$	**901.1511**	**901.4856**
Ovatoxin-f	OVTX-f	$C_{131}H_{227}O_{52}N_3$	905.1633	905.4977
Ovatoxin-g	OVTX-g	$C_{129}H_{223}O_{51}N_3$	890.4879	890.8223
Ovatoxin-h	OVTX-h	$C_{129}H_{225}O_{51}N_3$	891.1598	891.4942
Ovatoxin-i	OVTX-i	$C_{131}H_{225}O_{53}N_3$	909.8230	910.1575
Ovatoxin-j_1	OVTX-j_1	$C_{131}H_{225}O_{54}N_3$	915.1547	915.4891
Ovatoxin-j_2	OVTX-j_2	$C_{131}H_{225}O_{54}N_3$	915.1547	915.4891
Ovatoxin-k	OVTX-k	$C_{131}H_{225}O_{55}N_3$	920.4863	920.8208
Isobaric palytoxin	IsobPLTX	$C_{129}H_{223}O_{54}N_3$	906.4828	906.8172
**OVTX-a Degradation product**	**OVTX-a -1H_2O**	$C_{129}H_{221}O_{51}N_3$	**889.8160**	**890.1504**
OVTX-a Degradation product	OVTX-a -6H_2O	$C_{129}H_{211}O_{46}N_3$	859.7984	860.1328
OVTX-a Degradation product	OVTX-a -8H_20	$C_{129}H_{207}O_{44}N_3$	847.7913	848.1258
OVTX-d/e Degradation product	OVTX-d/e -6H_20	$C_{129}H_{211}O_{47}N_3$	865.1300	865.4645
OVTX-d/e Degradation product	OVTX-d/e -7H_20	$C_{129}H_{209}O_{46}N_3$	859.1265	859.4610
OVTX-d/e Degradation product	OVTX-d/e -8H_20	$C_{129}H_{207}O_{45}N_3$	853.1210	853.4574
OVTX-d/e Degradation product	OVTX-d/e -9H_20	$C_{129}H_{205}O_{44}N_3$	847.1195	847.4539
Isobaric PLTX Degradation product	IsobPLTX -7H_20	$C_{129}H_{209}O_{47}N_3$	864.4581	864.7926
Isobaric PLTX Degradation product	IsobPLTX -8H_20	$C_{129}H_{207}O_{46}N_3$	858.4546	858.7891
**Truncated OVTX-a (COOH)**		$C_{123}H_{213}O_{51}N$	**853.7931**	**854.1275**
**Putative truncated OVTX-a (Ethyl Ester)**		$C_{125}H_{217}O_{51}N$	**863.1368**	**863.4713**
Unknown PLTX-like compound			909.8352	910.1696
**Unknown PLTX-like compound (1)**			**859.1222**	**859.4565**
**Unknown PLTX-like compound (2)**			**853.1182**	**853.4527**

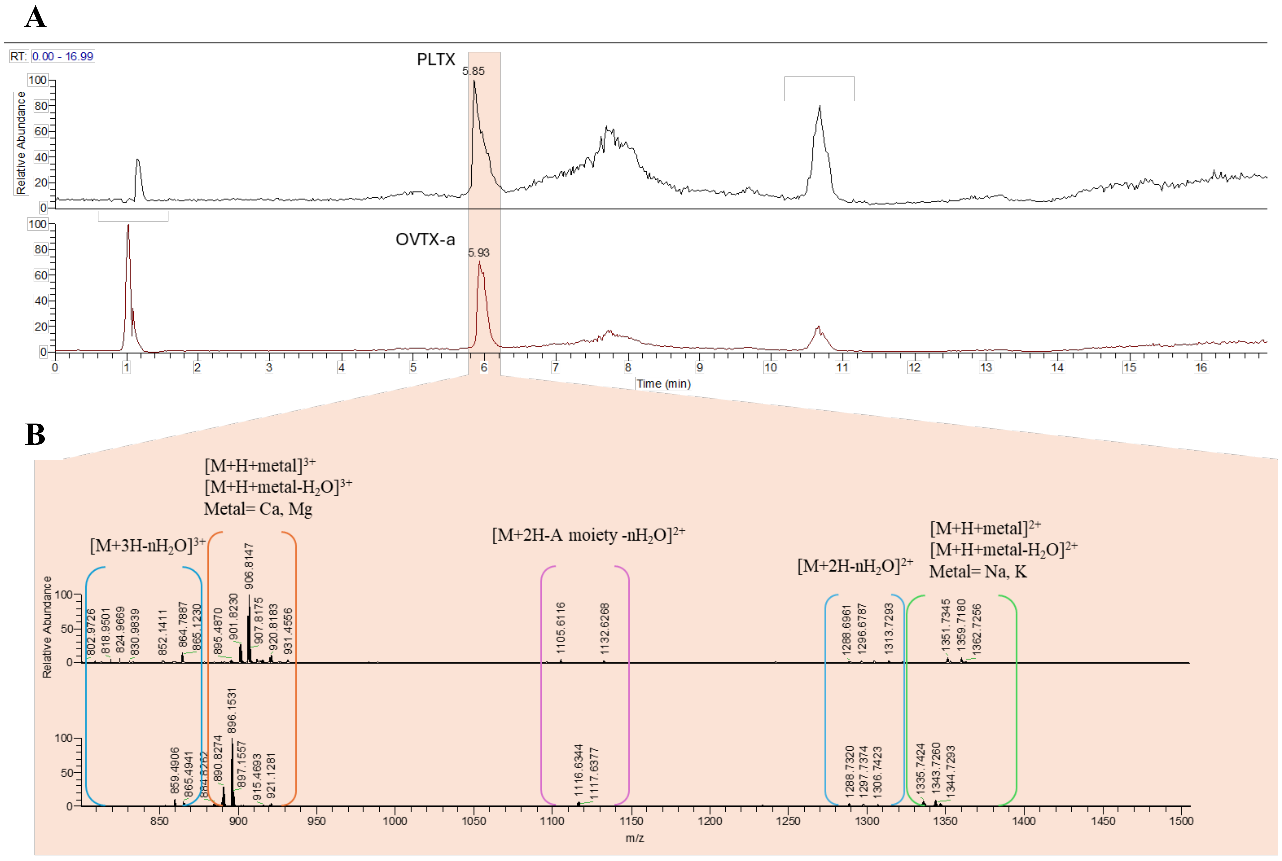

Fig. S3 (A) Total Ion Chromatogram (TIC) of PLTX standard (lot n° 16115131) and OVTX-a isolated from the *O.* cf. *ovata* NIES 3351 (Sample ID 5a) in EtOH:W, (1:1, v/v) obtained using fast gradient elution (Miele et. al. 2024) and (B) Full scan HRMS spectra (*m/z* 800-1500) associated to the chromatographic peaks of PLTX (above) and OVTX-a (below) with indication of the ion assignment of the triply- and doubly-charged ion clusters.

Table S2 Quantitative results of the OVTX-a end-product (Sample ID 5a). Extracted Ion Chromatograms (XIC) were obtained by summing the monoisotopic and most abundant peaks of the $[M+H+Ca]^{3+}$, $[M+H+Na]^{2+}$, and $[M+H+K]^{2+}$ ion clusters for PLTX and OVTX-a.

Compound	ION	XIC monoisotopic, most abundant ion (*m*/*z*)	Peak Area	Dilution factor	Conc. µg/ml
**PLTX standard in EtOH:W 1:1**	$[M+H+Ca]^{3+}$	906.4802, 906.8147	3816134	1	1
	$[M+H+Na]^{2+}$	1351.2333, 1351.7345			
	$[M+H+K]^{2+}$	1359.2164, 1359.7179			
**OVTX-a Sample ID5a_Dil 1:10**	$[M+H+Ca]^{3+}$	895.8189, 896.1531	9817944	10	**25.7**
	$[M+H+Na]^{2+}$	1335.2419, 1335.7424			
	$[M+H+K]^{2+}$	1343.2244, 1343.7260			
